# Isolated Internuclear Ophthalmoplegia as an Embolic Complication of Transcatheter Aortic Valve Implantation

**DOI:** 10.7759/cureus.32292

**Published:** 2022-12-07

**Authors:** Simon Thebault, Jodi Warman-Chardon, Kathleen O'Connell, William D Miller, Pierre R Bourque

**Affiliations:** 1 Neurology, The Ottawa Hospital, Ottawa, CAN; 2 Emergency Medicine, The Ottawa Hospital, Ottawa, CAN; 3 Neuroradiology, The Ottawa Hospital, Ottawa, CAN

**Keywords:** horizontal diplopia, internuclear ophthalmoplegia, transcatheter aortic valve implant, mri, cardioembolic stroke

## Abstract

An 83-year-old male developed horizontal diplopia immediately following elective transfemoral transcatheter aortic valve implantation (TAVI). On right gaze, left eye adduction was impaired while there was horizontal nystagmus of the abducting right eye, representative of internuclear ophthalmoplegia (INO). The remainder of the neurological examination was normal. Computer tomography (CT) imaging of the brain and CT angiogram of the head and neck were normal. Magnetic resonance imaging (MRI) of the brain showed five small foci of restricted diffusion affecting both the anterior and posterior circulation bilaterally. One such tiny infarct was seen in the left parasagittal upper pontine tegmentum and was attributed to his presentation. While all symptoms rapidly improved, minimal residual signs of INO were still detectable at the six-month follow-up. Isolated intra-nuclear ophthalmoplegia is a rare stroke syndrome and an unusual cardio-embolic complication of minimally invasive cardiac procedures. TAVI is an increasingly popular technique, although has been associated with a higher incidence of micro-embolic cerebrovascular events evident on MRI than surgical repairs. While the use of embolic protection devices has high-quality evidence in reducing the burden of these usually silent cerebrovascular events, their role in preventing long-term neurocognitive sequala has not been demonstrated.

## Introduction

We present a classical isolated syndrome of internuclear ophthalmoplegia (INO) in a patient with neuroimaging evidence of a more widespread embolic shower due to transcatheter aortic valve implantation (TAVI). While isolated INO is a rare stroke syndrome [1], micro-embolic cerebrovascular events evident on magnetic resonance imaging (MRI) in association with diagnostic or therapeutic angiographic interventions are seen in most patients [2]. Although usually 'clinically silent', the long-term neurocognitive sequelae of these events are a concern; the need for embolic protection devices in TAVI protocols remains an area of debate. Although there was an excellent symptomatic recovery of the INO, our case is a vivid reminder of the high frequency of these micro-embolic events, especially pertinent as TAVI gains traction as a predominant therapeutic option for aortic valvular repair.

## Case presentation

The inpatient neurology team was called on postoperative day 2 to assess an 83-year-old man who had developed horizontal diplopia immediately following elective TAVI. He reported that immediately after the procedure, he noticed that he had double vision especially worse with a distant gaze. The left transfemoral approach insertion of a SAPIEN 3 transcatheter valve (Edwards Lifesciences, Irvine, California) was successfully performed using heparin anticoagulation coverage to achieve an activated clotting time of >250 seconds, followed by protamine reversal at the end of the procedure. He was known to have an incomplete left bundle block before the procedure, but intraoperatively, he developed a transient complete heart block requiring transvenous pacing via the internal jugular vein, subsequently replaced with a single chamber permanent pacemaker for post-procedure 2:1 atrioventricular block and complete left bundle branch block. In the recovery room, he required intravenous fluids and a low-dose norepinephrine infusion for a total of six hours to maintain a blood pressure above 90 mmHg.

In addition to severe aortic stenosis (aortic valve area 0.68 cm^2^, pressure gradient of 68.3 mmHg), past medical history was revealing of a peri-procedural stroke 13 years prior immediately following percutaneous coronary intervention for angina and triple vessel coronary artery disease. The details of this event, performed abroad, were not available. According to available collateral history, this resulted in two weeks of aphasia followed by complete resolution. Additional comorbidities included hypertension, dyslipidemia, type 2 diabetes, chronic lymphocytic leukemia, and congestive heart failure New York Heart Association class II. He was a former smoker with a 35-pack-year history. 

**Figure 1:**
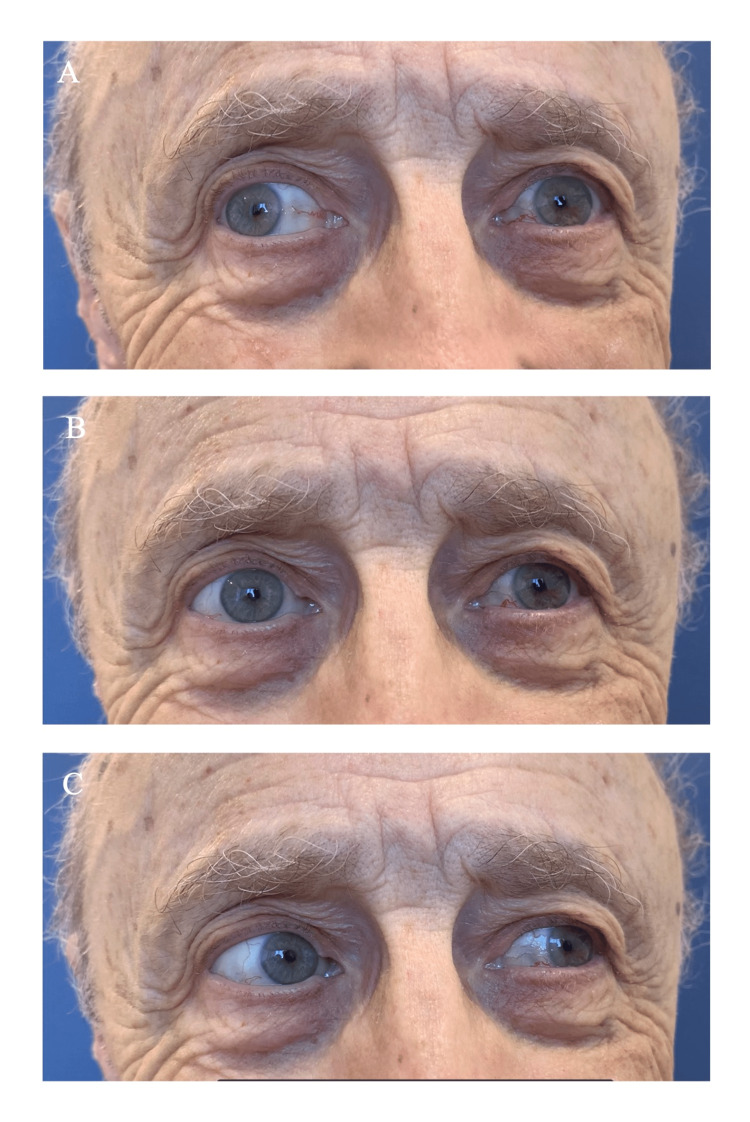
Left internuclear ophthalmoplegia Impaired left eye adduction on right gaze (Figure 1A); otherwise normal horizontal eye movements looking straight (Figure 1B) and left (Figure 1C), in keeping with left internuclear ophthalmoplegia.

**Figure 2:**
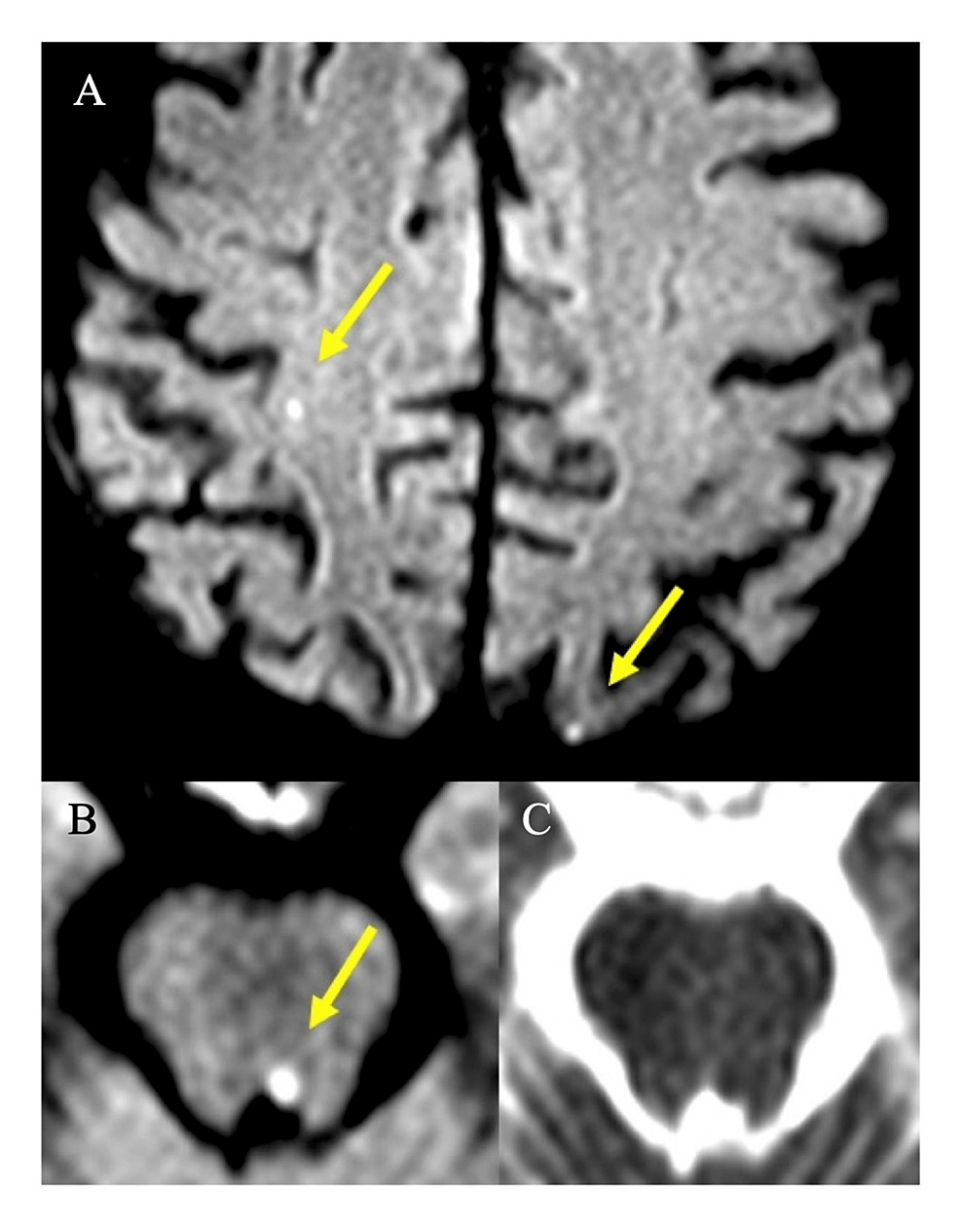
Cerebral microemboli on MRI following TAVI Diffusion-weighted imaging at b1000 showing small infarcts (arrows) in the right parietal left occipital lobes (Figure 2A) and left medial longitudinal fasciculus (Figure 2B). Apparent diffusion coefficient of the left medial longitudinal fasciculus stroke (Figure 2C). TAVI: transcatheter aortic valve implantation

Neurological examination revealed impaired left eye adduction on the right gaze with horizontal nystagmus of the abducting right eye (Figure 1 and Video 1). Cognition, language, motor, sensory, coordination, and gait examination were normal. Computer tomography (CT) imaging of the head was unrevealing, and a CT angiogram of the head and neck showed no evidence of significant stenosis or occlusion. There was mild to moderate atherosclerosis of the intradural segment of the left vertebral artery. With a clinical suspicion of INO of ischemic origin relating to the TAVI procedure, an MRI of the brain was obtained. This showed five small foci of restricted diffusion in both the anterior and posterior circulation bilaterally (representative examples shown in Figure 2A) in addition to the culprit tiny infarct in the left parasagittal upper pontine tegmentum (Figure 2B). Additional stroke workup revealed a hemoglobin A1c of 7.2% and low-density lipoprotein of 1.7 mmol/L.

**Video 1:** Left internuclear ophthalmoplegia Impaired left eye adduction on right gaze; otherwise normal horizontal eye movements looking straight and left, in keeping with left internuclear ophthalmoplegia.

Medical stroke management consisted of dual antiplatelet therapy for three weeks, followed by aspirin monotherapy, in addition to secondary prevention consisting of minor modifications to the antidiabetic regime. For diplopia, he was treated symptomatically with a left eye patch and occupational therapy referral. At the six-month follow-up, he was symptomatically well, with no residual diplopia or related symptoms otherwise. However, on examination, he had persistent subtle jerk nystagmus of the right eye on the right gaze, which was felt to be a residual deficit of the INO.

## Discussion

Internuclear ophthalmoplegia (INO) is a distinct clinical syndrome, readily diagnosed by the combination of ipsilateral adduction weakness and contralateral abduction nystagmus. In a young patient, unilateral or bilateral INO is often suggestive of multiple sclerosis. In an older patient, INO is most likely associated with ischemia in the distal territory of paramedian pontine tegmental arteries [3]. INO has been estimated to be the main presenting feature of 0.5% of ischemic strokes [4]. A well-referenced Korean case series of strokes causing INO as the predominant feature of brainstem infarction has provided a detailed clinical-neuroimaging correlation [1]. The main initial clinical symptom was horizontal diplopia, maximal with gaze directed opposite to the side of impaired adduction. Vertigo and dizziness were also commonly reported. On examination, convergence was preserved in about 60% of patients and skew deviation was noted in 40%. The range of embolic or atherothrombotic vascular occlusions associated with isolated INO was, however, wide, including lesions of distal penetrating basilar branches, basilar trunk, superior cerebellar, and posterior cerebral arteries [1].

As was seen in our patient, isolated ischemic INO has an excellent functional prognosis, whereas INO with additional deficits implicating other brainstem nuclei or long tracts is likely to recover less rapidly or completely. A very similar case of periprocedural isolated INO, in the setting of percutaneous cardiac intervention for myocardial infarction, has been published [4].

In our patient, there were compelling reasons to implicate a microembolic shower as the definitive etiologic factor. Indeed, there was no evidence of significant atherosclerotic occlusion upon imaging of the posterior circulation, and the MRI showed several similar, concurrent, dot-sized ischemic cortical infarcts involving both anterior and posterior circulation distal branches bilaterally. All these additional minute infarcts were clinically silent.

Modern neuroimaging techniques, notably the advent of diffusion-weighted imaging (DWI), have made it possible to better quantify the incidence of cerebrovascular complications in the setting of interventions ranging from angioplasty and stenting of carotid or coronary arteries to simple diagnostic aortic angiography or endovascular valvular repair. The majority of DWI lesions noted after such procedures are asymptomatic, by virtue of their small volume and involvement of non-eloquent brain regions. It has, however, been postulated that routine clinical assessments are mostly targeted at detecting obvious motor, visual, oculomotor, or specific cognitive domains such as language, praxis, and memory. More subtle neuropsychological deficits are not captured by such assessments and an accumulation of “silent” brain infarcts may contribute to neuropsychologic deficits in this patient population [5].

TAVI is the treatment of choice for symptomatic aortic valve stenosis in older and more comorbid patients for whom surgical repair is considered too invasive [6] and is increasingly used in younger and lower-risk groups that previously would be treated with conventional valvular surgery [7]. While TAVI operative approaches vary (e.g. transfemoral, transaxillary/subclavian, transapical), the overall incidence of clinically significant cerebrovascular events associated with the procedure is in the range of 3-4% [8]. However, DWI has detected new silent cerebral lesions in between 60% and 90% of patients undergoing TAVI [2,9,10], significantly higher than the rate in patients undergoing surgical repair. Additionally, high-intensity signals (HITS) have been detected by transcranial Doppler studies in virtually all of these patients, with the greatest frequency at the time of valve positioning and implantation [11]. In one randomized trial of high-risk patients undergoing TAVI with or without embolic protection [12], the use of such a device resulted in a 60% seven-day reduction in the median number of these lesions and a 54% reduction in mean total lesion volume. Although the long-term neurocognitive implications of clinically silent infarcts is a debatable [10], the possible neurocognitive sequelae of these lesions remain an area of concern, especially with the availability of effective mitigation devices and the adoption of TAVI.

## Conclusions

TAVI is an increasingly popular technique that is frequently associated with microembolic cerebrovascular events. Isolated internuclear ophthalmoplegia is an unusual complication. While our patient had a very favorable outcome, this case highlights the potential role of embolic protection devices. These devices reduce the burden of TAVI-associated cerebrovascular events and may have a role in preventing clinical strokes and neuropsychological disability.
